# Exogenous activated NK cells enhance trafficking of endogenous NK cells to endometriotic lesions

**DOI:** 10.1186/s12865-015-0105-0

**Published:** 2015-08-29

**Authors:** Mary Lourdes Montenegro, Rui Alberto Ferriani, Per H. Basse

**Affiliations:** Department of Gynecology and Obstetrics of Faculty of Medicine of Ribeirão Preto, University of São Paulo, Ribeirao Preto, SP Brazil; Department of Immunology of University of Pittsburgh, Pittsburgh, PA USA

**Keywords:** Endometriosis, NK cells, Activated NK cells, A-NK cells, Treatment

## Abstract

**Background:**

Endometriosis is defined as the presence of endometrial glands and stroma at ectopic locations. Although the prevalence of endometriosis is as high as 35 %–50 %, its pathogenesis remains controversial. An increasing number of studies suggest that changes in immune reactivity may be primarily involved in the development of endometriosis development. In this sense, it has been strongly suggested that a fundamental part of immunologic system, the natural killer cells (NK cells), are an important part of this process. NK cells, a component of the innate immune system, have been extensively studied for their ability to defend the organism against infections and malignancy. Recent studies have shown that IL-2-activated NK (A-NK) cells are able to attack and destroy tumors in lungs and livers of mice, demonstrating the therapeutic potential of these cells. Similarly to metastatic tumor cells, endometrial cells are able to adhere, infiltrate and proliferate at ectopic locations. Therefore, in this study, we evaluated the ability of adoptively transferred and endogenous NK cells to infiltrate endometriosis lesions.

**Methods:**

As NK cells donors were used C57BL/6 B6. PL- Thy 1.1 female mice. As uterine horns donors were used C57/BL6+GFP female mice and as endometriosis recipients C57BL/6 Thy1.2 female mice. Endometriosis induction was made by injection of endometrial tissue fragments. After 4 weeks, necessary for endometriosis lesions establishment the animals were divided in 3 experimental groups with 10 animals each. Group 1 received i.v doses of 5x106 A-NK in 200μl RPMI; Group 2 received i.p dose of 5x106 A-NK in 200μl RPMI and Group 3 received i.p dose of IL2 (0.5 mL RPMI containing 5.000U of IL2).

**Results:**

Our data show that exogenous A-NK cells injected via ip combined with endogenous A-NK cells seems to be the most efficient way for activated NK cells track and infiltrate endometriosis.

**Conclusion:**

For the first time, it was shown that both endogenous as exogenous A-NK cells are able to track, migrate and infiltrate endometriosis lesion. This seems to be a promising result, and if confirmed the efficiency of A-NK cells in killing endometriosis lesions, maybe in the future we could use this approach as an alternative treatment for women with endometriosis.

## Background

Endometriosis is cracterized by presence of endometrial glands and stroma in ectopic locations, as pelvic peritoneum, ovaries, and rectovaginal septum, affecting 6 % to 10 % of women in reproductive age [1]. Endometriosis can cause dysmenorrhea, dyspareunia, chronic pelvic pain and infertility [1]. The prevalence of endometriosis in women experiencing pain, infertility, or both is as high as 35 %–50 % [2]. The pathogenesis of endometriosis remains controversial. Currently, an increasing number of studies have addressed whether changes in immune reactivity may facilitate development of endometriosis [3–5]. In this sense, it has been strongly suggested that a fundamental part of immunologic system, the natural killer cells (NK cells), are an important part of this process [6, 7].

NK cells are large granular lymphocytes, representing about 5 % to 15 % of peripheral blood lymphocytes [8]. NK cells are an important component of the innate immune system and have been extensively studied for their ability to defend the organism against infections and malignancies [9]. NK cells kill their targets by direct lysis or by the release of cytokines and chemokines [10]. These reactions are carefully balanced and the participation of cytokines such as interferons α and β and interleukin 2 (IL-2) are essential [11]. In addition to increasing the traffic of NK cells to sites of injury, IL-2 activation has a strong proliferative effect on NK cells [3]. Studies have shown that NK cells activated by IL-2 (Adherent NK cells or A-NK cells) are able to infiltrate and destroy tumors in lungs and livers of mice [10, 11], suggesting that activated NK cells may be of therapeutic importance in the setting of cancer. Recently, a study demonstrated that activation of leucocytes with IL-2, induced a persistent reduction of endometriosis lesions in female rats [12]. On this basis, is possible that IL-2 activated NK cells could form the basis of a new treatment alternative for patients with endometriosis.

## Results

Our analyses show that exogenous A-NK cells injected via the i.v or i.p routes very efficiently infiltrate endometriosis lesions (Figs. 3, 4). In non-treated animals, endogenous NK cells are only found in very low numbers in endometriosis lesions (Fig. 5). However, in animals treated with A-NK cells plus IL-2 or with IL-2 alone, the density of endogenous NK cells in the endometriotic lesions increased at least 10 fold (Figs. 6, 7).

**Fig. 1:**
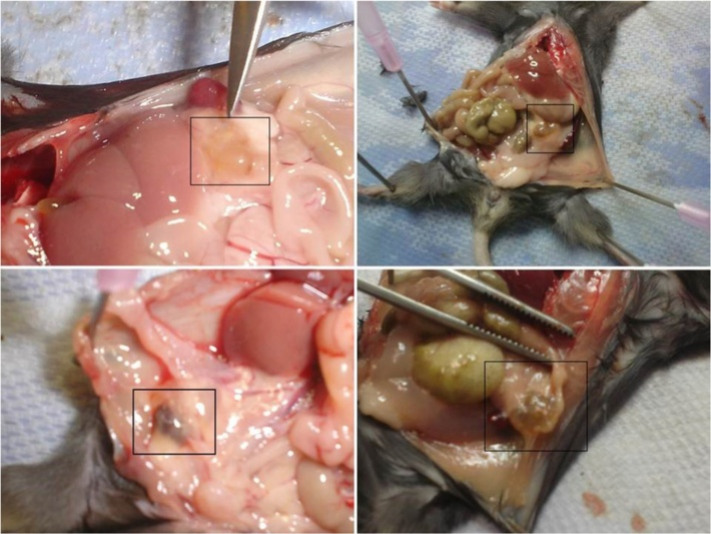
Experimental endometriosis lesions 4 weeks after injection of endometrial tissue. Donor mice: GFP transgenic C57BL/6; Recipient mice: wild-type (wt) C57BL/6. All pictures show endometriosis lesions from different animals

**Fig. 2:**
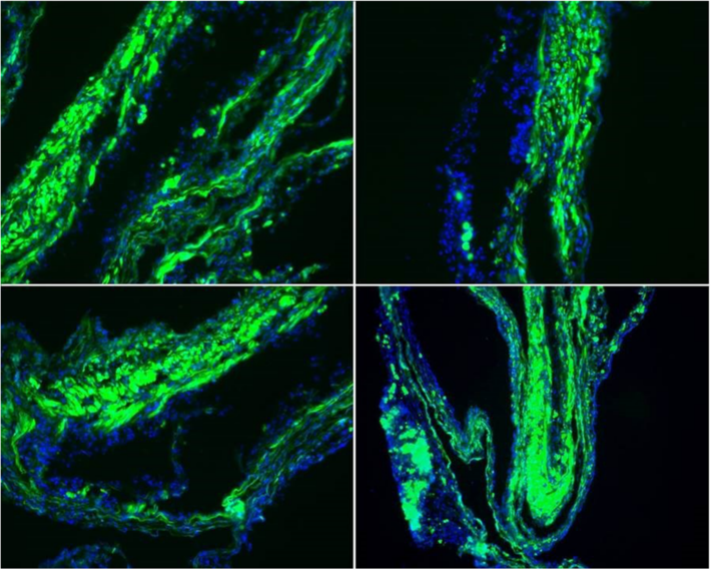
GFP-positive endometriosis lesions. Experimental endometriosis lesions were induced by i.p injection of fragments of endometrium from GFP transgenic C57BL/6 mice. The lesions were removed 4 weeks later, fixed in 4 % paraformaldehyde, frozen and sectioned. Sections were stained with Hoechst 33342 to reveal cell nuclei. The endometriosis tissue is strongly positive for GFP, proving that the tissue is of donor origin. Image resolution- 20X. All micrographs show endometriosis lesions from different animals

**Fig. 3:**
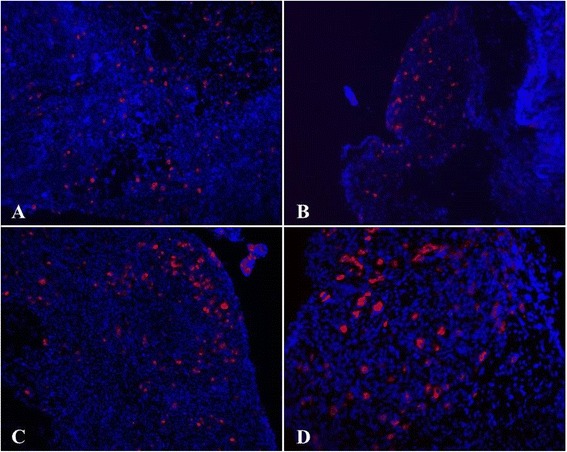
Exogenous A-NK cells injected intravenously localize at sites of endometriosis lesions. Fresh frozen endometriosis tissue was sectioned and stained with PE anti-CD90.1 to reveal the Thy1.1+, adoptively transferred A-NK cells in the Thy1.2+ recipients. Sections were stained with Hoechst 33342 to reveal cell nuclei. Image resolution 20X. All micrographs (**a**-**d**) show endometriosis lesions from different animals

**Fig. 4:**
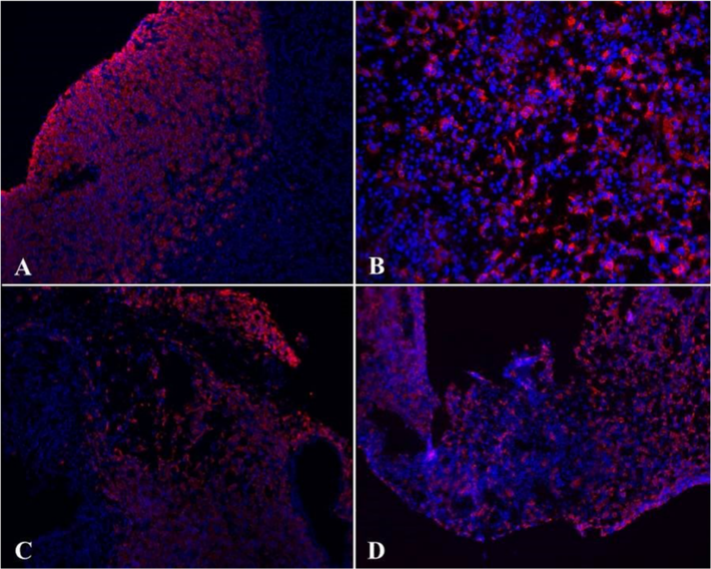
Exogenous A-NK cells injected intraperitoneally localize at sites of endometriosis lesions. Fresh frozen endometriosis tissue was sectioned and stained with PE anti-CD90.1 to reveal the i.p. injected, Thy1.1+ A-NK cells in the Thy1.2+ recipients. Sections were stained with Hoechst 33342 to reveal cell nuclei. Image resolution 20X. All micrographs (**a**-**d**) show endometriosis lesions from different animals

**Fig. 5:**
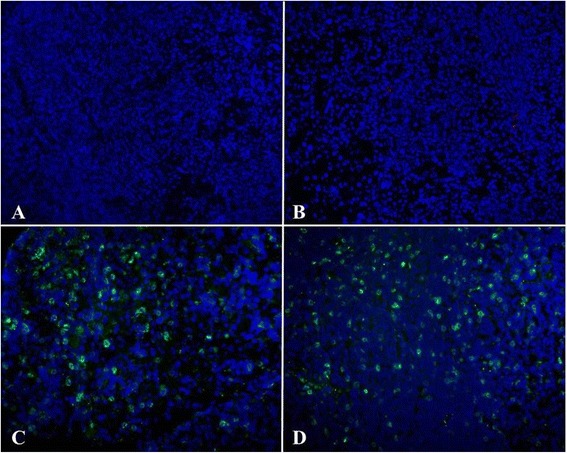
IL-2 treatment augments infiltration of endometriosis lesions by endogenous NK cells. Fresh frozen endometriosis tissue from animals receiving Peg-IL-2 only was sectioned and stained with anti-NKp46 (or isotype control) followed by Alexa 488-conjugated donkey anti-goat antibody to identify endogenous NK cells Sections were also stained with Hoechst 33342 to reveal cell nuclei. Image resolution 20X. (**a**-**b**) Isotype control plus Alexa 488 donkey anti-goat. (**c**-**d**) Staining with anti-NKp46 plus Alexa 488 donkey anti-goat antibody reveals a substantial infiltration of the lesions by endogenous NK cells. Image resolution 20X

**Fig. 6:**
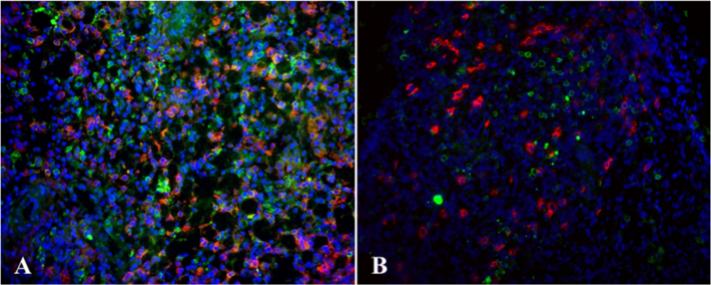
Infiltration of endometriosis lesions by endogenous NK and exogenous A-NK cells. Sections from endometriosis lesions were double-stained with PE anti-Thy1.1 antibody and anti-NKp46 antibody followed by Alexa 488-conjugated donkey anti-goat antibody to identify adoptively transferred A-NK cells (Thy1.1+/ NKp46+) and endogenous NK cells (Thy1.1-/NKp46+). Slides were stained with Hoechst 33342 (for nuclei). Image resolution 20X. (**a**) Endometriosis lesion from animal that received exogenous A-NK cells i.p. (**b**) Endometriosis lesion from animal that received exogenous A-NK cells by the i.v. route

**Fig. 7:**
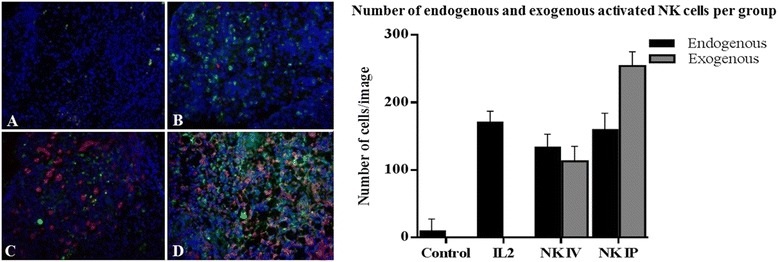
Density of endogenous and exogenous NK cells at sites of endometriosis. Endometriosis tissue was cut and stained as described in the legend to Fig. 6. (**a**) Endometriosis lesion from control animal (no treatment) (**b**) Endometriosis lesion from animal that received PEG-IL2 only. (**c**) Endometriosis lesion from animal that received i.v. A-NK cells by the i.v. route. (**d**) Endometriosis lesion from animals that received i.p. injection of A-NK cells. Image resolution 20X. Slides were stained with Hoechst 33342 (for nuclei). (**e**) Exogenous and endogenous NK cells were identified as PE-positive and Alexa 488-positive, PE-negative cells, respectively, and counted. Results are presented as mean number of cells/field of vision ± SD. Endogenous NK cells (black columns), exogenous A-NK cells (gray columns)

## Discussion

Endometriosis affects almost 10 % of women in the reproductive age. The pathogenesis of endometriosis remains controversial. However, many recent studies have implicated the immune system and especially NK cells in endometriosis development. On this basis, NK cells, a fundamental part of immunologic system seems to play an important role in endometriosis development. Several of these studies have reported a decrease in cytotoxicity of NK cells in the peritoneal fluid from women with endometriosis [12, 13, 18]. Lack of NK cell cytotoxicity could, at least in part, facilitate the attachment of endometriotic cells at ectopic sites [6]. However, the reason for the decrease in cytotoxicity of peritoneal fluid NK cells remains to be clarified, but only few studies have assessed the role of NK cells in target endometriosis lesions. Velasco and Cols found that treatment of female rats with endometriosis with IL-2 (two doses given by the i.p. route) resulted in the activation of leucocytes and a significant reduction in the size of endometriotic lesions compared to the untreated group [12]. However, when they look for activated NK cells, it seems to be in low number compared with control. Differently, in our study we show that when activated by IL2, endogenous NK cells starts to migrate to the lesion becoming able to infiltrate it and that this effect seem to be potentiate when exogenous A-NK were injected via i.v or i.p. This important evidence shown at least in part that NK cells seem really play an important role on pathogenesis of endometriosis. We believe that ours findings were different from those found for Velasco’s, first due the difference in experimental model, and second due the difference in protocols accessed by each study. While we collected tissue three days after A-NK cells induction, the other study wait 3 months to collect the simples, maybe this long time has influenced in activity and quantity of NK cells. On the other hand, due our short-term protocol, was not possible observe changes in lesions size. Despite of this, for the first time we show that activated NK cells are able to track and infiltrate endometriosis lesions and the best way to promote this effect is through combination of endogenous A-NK cells and exogenous A-NK cells injected via i.p.

## Conclusions

Our results show at least in part that exogenous activated NK cells enhance trafficking of endogenous NK cells to endometriotic lesions. It seems to be a very promising result, and if confirmed that A-NK cells are efficient in killing endometriosis lesions, maybe in the future we could use this approach as an alternative treatment for women with endometriosis. Therefore, we believe it is necessary understand better decreasing of cytotoxicity of NK cells in peritoneal fluid of women with endometriosis and why endogenous NK cells seems not to be able to attack endometriosis and also if exogenous A-NK cells despite infiltrate the lesions, are really able to kill endometriosis.

## Methods

The study was performed at University of Pittsburgh Cancer Institute and approved by University ethics committee under the Protocol N^o^ 12091050.

Splenocytes from C57BL/6 B6. PL- Thy 1.1 female mice were used for production of A-NK cells. As uterine horn donors we used C57/BL6 + GFP female mice (transgenic for green fluorescent protein) (Fig. 2) and endometriosis was established in C57BL/6 (Thy1.2) female mice. All mice were 8-12 weeks of age.

Endometriosis was induced by i.p. injection of endometrial tissue fragments, according Wieser et al [13]. To stimulate proliferation of endometrial tissue, GFP donor mice received estradiol valerate subcutaneously (100 μg/kg dissolved in corn oil) one week before harvest of uterine tissue. Following harvest, uterine horns were opened and placed in a sterile Petri dish with PBS [15]. The endometrium was detached from the uterine muscle and finely chopped with a scalpel [16, 17]. Finally, the endometrial fragments were suspended in 0.6 ml PBS and injected i.p into recipient mice using an 18 gauge disposable needle [13]. Each recipient mouse received an equal quantity of tissue (~40 mg). Recipient mice also received 100 μg/kg estradiol valerate subcutaneously once a week, starting one week before receiving the endometrial tissue. This procedure was performed in order to synchronize their estrous cycles. After 4 weeks, GFP-positive endometriosis lesions were established (Fig. 1) and the experimental protocol was started [18].

### Experimental protocols

Animals were divided in 3 experimental groups with 10 animals each. Group 1 received i.v doses of 5 × 10^6^ A-NK in 200 μl RPMI; Group 2 received i.p dose of 5 × 10^6^ A-NK in 200 μl RPMI; Group 3 received i.p dose of IL2 (0.5 mL RPMI containing 30.000 IU/mL of pegylated (PEG-) rhIL2). To support the transferred A-NK cells, animals from group 1 and 2 received i.p. injections of 0.5 mL RPMI containing 30.000 IU/mL of pegylated (PEG-) rhIL2 at 12 hour intervals for 3 days. Group 3 received the same amount of Peg-IL-2 (IL-2 control), whereas control group received injections of 0.5 ml RPMI without IL-2 [14].

### A-NK cells preparation

Spleens were removed aseptically from C57BL/6 B6. PL- Thy 1.1 female mice and a single-cell suspension was prepared in RPMI1640. Erythrocytes were lysed by incubation with ammonium chloride-potassium buffer at room temperature for 3 min and the spleen cells were subsequently washed twice in RPMI1640. Cells were transferred to T150 plastic flasks (Falcon, B&D, Franklin Lakes, NJ, USA) and cultured at 37 °C in an atmosphere of 5 % CO_2_ in 50 ml of RPMI1640 supplemented with 5 % heat inactivated fetal calf serum and 5 % normal human serum, 10 ml/l non-essential amino acids (Life Technologies), 50 mM 2-mercaptoethanol, 2 mM glutamine, 20 mM Hepes buffer, 0.8 g/l streptomycin and 1.6x105u/l penicillin, hereafter referred to as complete medium (CM). Cells were stimulated with 6.000U/mL of human recombinant IL-2. After 3 days of incubation, CD8-positive cells were magnetically removed following incubation of the cell culture with rat anti-CD8 antibody (ATCC, TIB-105) and subsequently with anti-rat coated magnetic beads (Dynal Biotech, Lake Success, NY, USA). The CD8-depleted cells were resuspended in fresh CM containing 6000 IU/mL IL-2 to a final concentration of 1x10^5^cells/ml and returned to culture flasks. After an additional 3 days of culture, non-adherent cells were decanted and the plastic-adherent cells were harvested after a brief treatment with 0.02 % EDTA and washed twice in RPMI1640 before use [11]. Routinely, these A-NK cells were 95 % Thy1.1, 95 % asGM1, 90 % NK1.1, 2 % CD8 and 2 % CD4 [11, 14].

### Adoptive transference of A-NK cells

A-NK cells were adjusted to appropriate concentrations and injected into C57BL/6 Thy1.2 mice via lateral tail vein (i.v.) or into the peritoneal cavity (i.p.). To support the transferred A-NK cells, animals from group 1 and 2 received i.p. injections of 0.5 mL RPMI containing 30.000 IU/mL of pegylated (PEG-) rhIL2 at 12 hour intervals for 3 days. Group 3 received the same amount of Peg-IL-2 (IL-2 control), whereas group 4 received injections of 0.5 ml RPMI without IL-2.

### Accumulation of exogenous and endogenous NK cells in endometriosis lesions

At three days after injection of A-NK cells, animals were sacrificed and the lesions removed. The collected tissues were sectioned and fixed in ice-cold acetone for 5 minutes. Sections were stained with PE anti-rat CD90.1 to reveal the Thy1.1+, adoptively transferred A-NK cells in the Thy1.2+ recipients. Sections we also stained with anti-NKp46 antibody followed by Alexa 488 donkey anti-goat antibody to reveal endogenous NK cells (NKp46+/Thy1.1-). Before coverslipping, sections we stained with Hoechst 33342 to reveal all nuclei.

The density of immune-stained cells were determined by image analysis (MetaMorph ®) [14].

### Statistical analysis

To compare all study groups, analysis of variance (ANOVA) was performed.

## Abbreviations

NK cells: Natural killer cells
IL2: Interleukin 2
i.v: Intravenous
i.p: Intraperitoneal
PEG-IL2: Human recombinant IL2 complexed to polyethylene glycol
GFP: Green Fluorescent Protein
A-NK cells: Activated Natural Killer Cells
